# Functional Analysis of Stress Resistance of *Bacillus cereus* SCL10 Strain Based on Whole-Genome Sequencing

**DOI:** 10.3390/microorganisms12061168

**Published:** 2024-06-08

**Authors:** Yanzhen Mao, Ye Yang, Fu Lin, Hanyu Chu, Lijie Zhou, Jiaojiao Han, Jun Zhou, Xiurong Su

**Affiliations:** 1 State Key Laboratory for Managing Biotic and Chemical Threats to the Quality and Safety of Agro-Products, Ningbo University, Ningbo 315832, China; mao2010111@163.com (Y.M.); 17815628500@163.com (Y.Y.); 18858047487@163.com (F.L.); 13462005107@163.com (H.C.); zhoulijie1113@163.com (L.Z.); hanjiaojiao@nbu.edu.cn (J.H.); suxiurong@nbu.edu.cn (X.S.); 2 School of Marine Science, Ningbo University, Ningbo 315832, China

**Keywords:** *Bacillus cereus*, stress resistance, bioinformatic analysis, gene function

## Abstract

A Gram-positive, rod-shaped, aerobic, motile, and spore-forming bacterium, designated SCL10, was isolated from *Acaudina molpadioides* exposure to Co-60 radiation. In this study, whole-genome sequencing was performed to identify the strain as *Bacillus cereus* and functional characterization, with a focus on stress resistance. The genome of the *B. cereus* SCL10 strain was sequenced and assembled, revealing a size of 4,979,182 bp and 5167 coding genes. The genes involved in biological functions were annotated by using the GO, COG, KEGG, NR, and Swiss-Prot databases. The results showed that genes related to alkyl hydroperoxide reductase (*ahpC*, *ahpF*), DNA-binding proteins from starved cells (*dps*), spore and biofilm formation (*spoVG*, *spo*0*A*, *gerP*), cold shock-like protein (*cspC*, *cspE*), ATP-dependent chaperone (*clpB*), and photolyase, small, acid-soluble spore protein (SASP) and DNA repair protein (*recA*, *radD*) could explain the stress resistance. These findings suggest that antioxidant activity, sporulation, biofilm formation, and DNA protection may be considered as the main resistance mechanisms under exposure to radiation in the *B. cereus* SCL10 strain.

## 1. Introduction

*Bacillus cereus* is a well-known foodborne pathogenic bacterium responsible for two types of food-associated gastrointestinal diseases, diarrheal and emetic, which are induced by toxins [1]. The diarrheal syndrome is caused by hemolysin BL (HBL), nonhemolytic enterotoxin (NHE), and cytotoxin K (CytK, also known as hemolysin IV); and the emetic syndrome is caused by cereulide [2]. Diarrhea and emesis caused by *B. cereus* are closely related to food infections, especially in rice, pasta, pastries, dairy, vegetables, and meat products. The diseases are generally mild and self-limiting, making it difficult for health authorities to determine the actual incidence [3]. The proportion of *B. cereus* infections is underestimated, and severe and fatal outbreaks have also been reported [4]. Despite the optimized combination of sterilization technologies such as heat, irradiation, and chemical reagents, *B. cereus* can still survive and pose a formidable challenge for food industries worldwide [5]. In addition to toxins and virulence factors, adaptability and resistance to the environment are also reasons for its pathogenicity and survivability [6].

In hostile environments, *B. cereus* has a self-protection system and develops resistance accordingly. As in most bacteria, once the level of reactive oxygen species (ROS) exceeds the capacity of endogenous antioxidant defense, the redox balance is disrupted and the oxidative stress system is activated [7]. Antioxidant defenses such as catalases, peroxiredoxins, and superoxide dismutases will promote anti-stress effects as one of the resistance strategies [8]. In addition, spores and biofilms have proven to be two forms of resistance mechanisms, leading to a high adhesion capacity on various substrates [9]. Meanwhile, resistance is affected by sporulation and biofilm-forming conditions. Spores and biofilms can both withstand extreme conditions, such as chemical agents, wetness, dry heat, low pH values, and radiation, an expanding contamination of food spoilage, and human diseases [10]. The resistance factors of spores include the outer layer, inner membrane, low water content, high levels of dipicolinic acid in the spore core, and DNA protection mechanism [11]. The appropriate conditions can prompt spores to return to vegetative cells through germination, which allows the application of milder inactivation procedures [12]. Biofilms are clusters of arranged bacteria that are attached to a surface and embedded in the self-produced matrix of the extracellular polymeric substance (EPS), where antibiotic resistance genes (ARGs) are easily transferred [13]. ARGs and genes associated with antioxidants, spores, and biofilms regulate the corresponding resistance.

Phenotypic resistance is known to have a genetic basis, which provides a reliable perspective on resistance mechanisms [14]. Any bacterium that survives adverse conditions of extreme temperature, radiation, and antibiotics would carry genes with different resistance levels and evolve multiple-stress resistance. Understanding the mechanisms underlying resistance contributes to cleaning procedures. Genomics presents a pathway for focusing on resistance genes and defense mechanisms. At present, whole-genome sequencing (WGS) of *B. cereus* is mostly employed in studies on routine surveillance, virulence, drug resistance mechanisms, and immune-related genes [15,16]. WGS can be used to analyze resistance in *B. cereus* at the molecular level. It is of interest to identify stress-related genes so that resistant bacteria that survive food sterilization would be better understood and more targeted sterilization methods could be applied.

The strain was isolated from the Co-60-induced watery sea cucumber (*Acaudina molpadioides*), designated the *B. cereus* SCL10 strain, which remained active and exposed to a high dose of Co-60 radiation. In this study, by combining pure culture and comparative genomics approaches, the WGS analysis was conducted to study stress-resistance genes and gene functions including metabolic pathways, protein functions, and virulence. Based on the predicted results, the sterilization and survival of *B. cereus* during food processing and preservation will be better understood. This study is expected to provide a genomic reference for resistance mechanism studies of *B. cereus*, contributing to food safety and public health.

## 2. Materials and Methods

### 2.1. Isolation and Culture of the Strain

The *B. cereus* SCL10 strain was isolated from the Co-60-induced watery sea cucumber (*A. molpadioides*), and preserved at Microbiology Laboratory, Ningbo University, China. The *B. cereus* SCL10 strain was cultured in a nutrient broth (NB) medium comprising peptone at 10.0 g/L, beef extract at 3.0 g/L, NaCl at 5.0 g/L, and additional NaCl at 15.0 g/L (simulated the growth environment of *A. molpadioides*), and an NB agar medium comprising peptone at 10.0 g/L, beef extract at 3.0 g/L, NaCl at 5.0 g/L, agar at 20.0 g/L, and additional NaCl at 15.0 g/L. The preserved strain was applied to the NB agar plate after gradient dilution, and incubated aerobically at 37. A single colony was picked and incubated into the NB at 37 °C and 150 revolutions per minute (rpm) for 12 h. The *B. cereus* pellet was obtained by centrifugation at 6000 rpm for 5 min and then washed twice with 0.10 mol/L phosphate buffer saline (PBS, pH 7.4). It was subsequently resuspended in the 0.10 mol/L PBS and prepared for the extraction of genomic DNA.

### 2.2. Genomic DNA Extraction and Whole-Genome Sequencing

According to the previous literature, genomic DNA was extracted with the SDS method [17,18]. The DNA of the *B. cereus* SCL10 strain was quantified using a Qubit 2.0 Fluorometer (Thermo Fisher Scientific, Waltham, MA, USA), and PCR products were purified. The sequencing library was generated with NEBNext Ultra II DNA Library Prep Kit for Illumina (NEB, Ipswich, MA, USA), and then was analyzed for size distribution by Agilent 2100 Bioanalyzer (Agilent, Santa Clara, CA, USA) and quantified by real-time PCR. The draft genome of the *B. cereus* SCL10 strain was sequenced with Illumina NovaSeq PE150 at LC-Bio Technology Co., Ltd. (Hangzhou, Zhejiang, China). The raw data were assembled with SOAP Denovo, SPAdes, and Abyss, filtering the low-quality reads out.

### 2.3. Genome Component Prediction

Genome component prediction included the prediction of the coding genes, non-coding RNAs, repetitive sequences, transposons, prophages, genomic islands, and clustered regularly interspaced short palindromic repeat (CRISPR) sequences. The GeneMarks program was used to retrieve the related coding genes and the RepeatMasker was used to search for repetitive sequences, the Tandem Repeats Finder (TRF) [19] to tandem repeats, the tRNAscan-SE to transfer RNA (tRNA) genes, the rRNAmmer to ribosome RNA (rRNA) genes, Basic Local Alignment Search Tool (blast) against the RNA families (Rfam) database [20] to small nuclear RNAs (snRNAs), the IslandPath-DIOMB program to the Genomics Islands, transposonPSI to the transposons, the PHAST to the prophages, and the CRISPR Finder to CRISPRs.

### 2.4. Genome Function Annotation

Eight databases were used to predict gene functions. It contained Non-Redundant Protein Sequence Database (NR), Kyoto Encyclopedia of Genes and Genomes (KEGG), Clusters of Orthologous Groups (COGs), Gene Ontology (GO), carbohydrate-active enzymes (CAZy), Transporter Classification Database (TCDB), protein families (Pfam), and Swiss-Prot Protein Sequence Database (Swiss-Prot), respectively [21]. A blast search of the whole genome of the *B. cereus* SCL10 strain was performed against the above eight databases. Meanwhile, we analyzed secondary metabolism gene clusters by the Antibiotics and Secondary Metabolite Analysis Shell (antiSMASH) [22]. Also, we used the Virulence Factors of Pathogenic Bacteria Database (VFDB) to study pathogenicity and used Comprehensive Antibiotic Research Database (CARD) to study antibiotic resistance genes [23].

### 2.5. Comparative Genomic Analysis

The comparative genomic analysis is applied among the *B. cereus* SCL10 strain and the other 19 strains (detailed strain information in Table S1), including core genes, specific genes, a synteny analysis, and a gene family phylogenetic tree. Genomic alignment between the sample genome and reference genomes was performed with the MUMmer and LASTZ. Based on the alignment results, the synteny analysis was among the *B. cereus* SCL10 strain and the most similar strain MH19. The gene family was constructed using blast and Hcluster_sg.

## 3. Results

### 3.1. Identification of B. cereus by WGS

**Figure 1:**
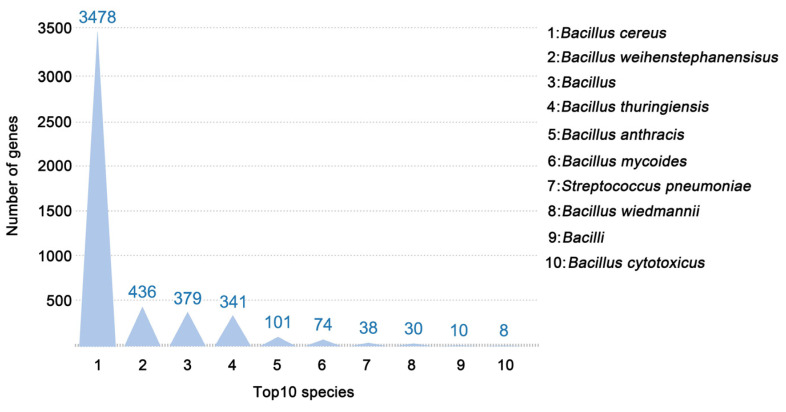
The species identification of genes annotated. The top 10 species with the highest matches were selected in the Non-Redundant Protein Sequence (NR) Database. The height of the bar represents the number of genes.

When cultured on the NB agar medium, the colonies of *B. cereus* were white, round, flat, and soft, with irregular margins, a slightly dry and rough surface, and a special feature of surface elevation (Figure S1). The optimal matching result for the genome showed that 4955 unigenes (refers to non-redundant gene sequences obtained from whole-genome sequencing) of the strain were annotated, accounting for 95.90% of the total coding genes in the NR database (Figure 1). A total of 3478 species-related unigenes were identified for the strain *B. cereus*.

### 3.2. Composition of the Overall B. cereus SCL10 Strain Genome

The *B. cereus* SCL10 strain genome consisted of 34 contigs (>500 bp) of 4,979,182 bp, with a GC% = 35.42. The Q20 and Q30 values were 97.51% and 93.25%, indicating good quality of the genome. The overall annotation information of the genome is listed in Table 1. Data validity information is provided in the Supplementary Materials (Figure S2). The assembled whole genome contained 5167 coding sequences (4955 protein-coding genes with functional assignment), and the total length was 4,167,474 bp, accounting for 83.70% of the genome. The main genome features of the *B. cereus* SCL10 strain included 33 transposons, 115 long terminal repeats, 25 long scattered repeats, 10 short scattered repeats, 12 prophages, and six CRISPRs. In addition, regarding non-coding RNAs, the genome encodes 89 tRNAs, 14 rRNAs, and six sRNAs.

### 3.3. General Functions of B. cereus SCL10 Strain Annotation

**Figure 2:**
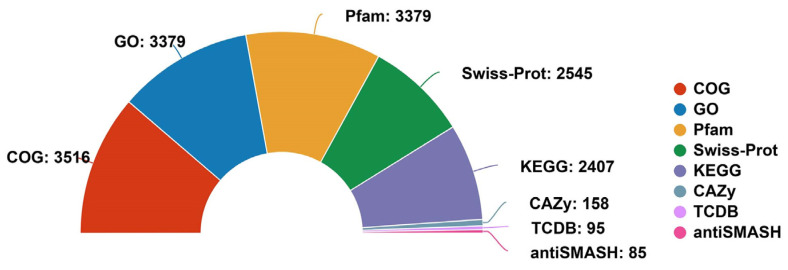
The general function annotation of the *B. cereus* SCL10 strain involving eight databases. The number of genes annotated varies across different databases, which can be compared with a total number of 5167 coding sequence genes. The eight databases are labeled in different colors and the numbers are gene counts. COG: Cluster of Orthologous Groups of Proteins, GO: Gene Ontology, Pfam: Protein Family, Swiss-Prot: Non-redundant High-quality Proteins, KEGG: Kyoto Encyclopedia of Genes and Genomes, CAZy: Carbohydrate-active Enzymes, TCDB: Transporter Classification Database, antiSMASH: Secondary Metabolism Gene Clusters.

**Figure 3:**
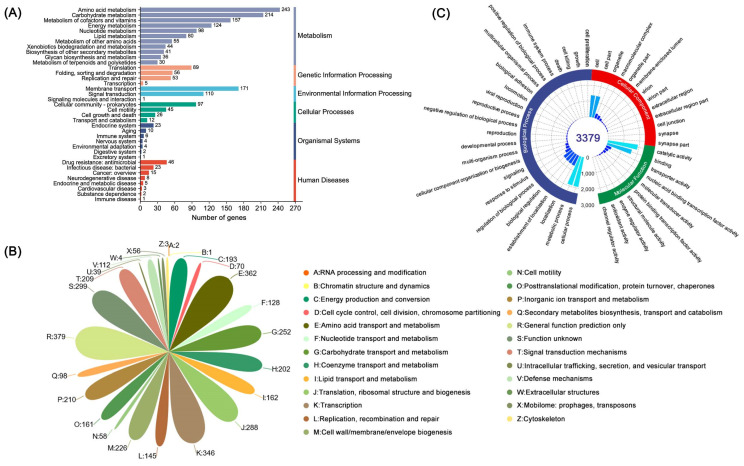
The different function classification of the *B. cereus* SCL10 strain. (**A**) KEGG annotation. The *X*-axis is the number of genes, and the *Y*-axis is the KEGG pathway. Different colors of the columns represent different categories, and the corresponding category names are on the right side. (**B**) COG annotation. Different letters and colors represent different classifications, and the numbers beside the petals represent the number of genes. (**C**) GO annotation. The three colors of the outer circle represent the three categories, which are used to distinguish biological processes, cell components, and molecular functions. The bars indicate the number of genes with different functions at all levels of the catalog.

Using the GO, COG, KEGG, Pfam, Swiss-Prot, and TCDB databases as a reference, various biological functions of genes and gene clusters were annotated in the *B. cereus* SCL10 strain genome. The number of genes found within each category and their sources are shown below (Figure 2). KEGG pathways were associated with 2407 unigenes, which were gathered into 199 metabolic pathways, including 243 unigenes in amino acid metabolic pathways, 214 in carbohydrate metabolic pathways, and 171 in membrane transport pathways of the top three pathways (Figure 3A). In addition, 53 unigenes in metabolic pathways for replication and repair were annotated. The detailed KEGG map showed 140 unigenes involved in ABC transporters (map02010), 88 in quorum sensing (map02024), and 30 in flagellar assembly (map02040) (Table S2).

A total of 68.05% of unigenes (3516) were represented in 26 classes by COG functional categories (Figure 3B). The protein functions of the *B. cereus* SCL10 strain were associated with cell wall/membrane/envelope biogenesis (M); post-translational modification, protein turnover, and chaperones (O); replication, recombination, and repair (L); and defense mechanisms (V), with 226, 161, 145, and 112 unigenes, respectively (Table S3). Defense mechanisms included organic hydroperoxide reductase (*ohrA*, *ohrR*, *osmC*), DNA-binding ferritin-like protein (oxidative damage protectant), alkyl hydroperoxide reductase subunit (*ahpC, ahpF*), and glutathione peroxidase, and the chaperone included ATP-dependent protease (*clpA*, *clpP*).

The GO database defines three major categories: the biological process, molecular function, and cellular component (Figure 3C). The biological process had the largest number and function types of annotated genes (7741), accounting for 48.68% of all annotation information, including biological regulation, response to a stimulus, signaling, and other biological processes related to adaptation. There were 371 responses to stimulus unigenes and 57 membrane-enclosed lumen unigenes. The two remaining categories were the molecular function and cellular component. The molecular function was associated with 337 unigenes involved in transporter activities, 28 in enzyme regulation, and 9 in antioxidant activities. Importantly, protein-encoding genes associated with general stress protein and ATP-dependent chaperone ClpB were found in the GO annotation results, covering 31 and 32 classes, respectively (Table S4).

**Figure 4:**
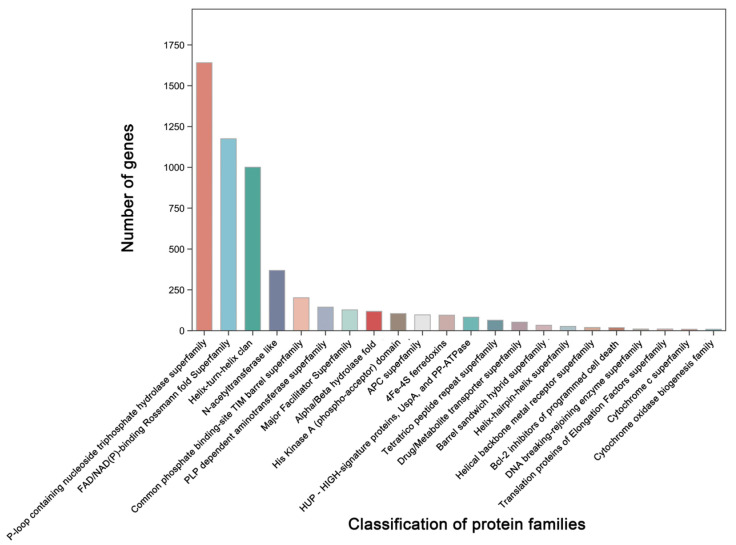
The classification and gene number of protein families of the *B. cereus* SCL10 strain. The top 20 protein families with the highest numbers and correlations with resistance were selected in the protein families (Pfam) database.

**Figure 5:**
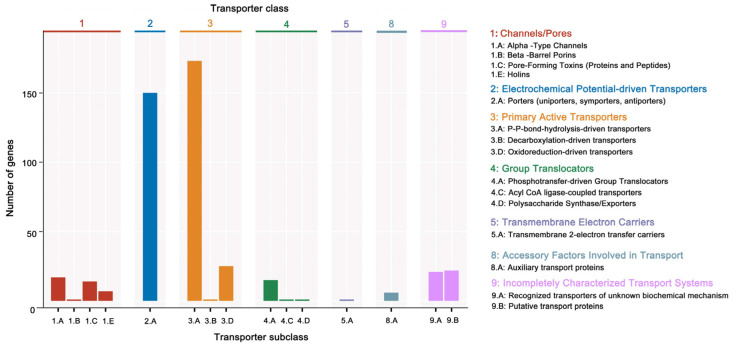
The TCDB annotation of the *B. cereus* SCL10 strain. The TC system is classified into 5 levels, each level corresponds to a letter or number in the TC number, and each letter or number represents a specific type of transport protein. Level 2 is a more specific subcategory below Level 1, and Level 3 is a transporter protein family classification. The horizontal coordinate indicates the number of Level 2.

Additionally, 3379 unigenes were associated with a protein family in the Pfam database, including 18 members of *bcl-2* inhibitors of programmed cell death and 10 members of the DNA breaking–rejoining enzyme superfamily (Figure 4, Table S5). A total of 2545 unigenes were annotated in the Swiss-Prot database, representing high-quality protein genes with low redundancy (Table S6). Furthermore, four types of secondary metabolism gene clusters of the *B. cereus* SCL10 strain were found: lassopeptide, siderophore, bacteriocin, and betalactone (Figure S3A). The classification of carbohydrate-active enzymes revealed 60 glycosyltransferases (GTs), 43 glycosidase hydrolases (GHs), 38 carbohydrate-binding modules (CBMs), and 17 carbohydrate esterases (CEs) (Figure S3B). The TCDB system, comprising 5 levels, revealed 199 major active transport proteins, 150 electrochemical potential-driven transport proteins, 39 channel/micropores, and other transport proteins (Figure 5, Table S7).

### 3.4. Genes Related to Flagellum and Enzymes in Genomic Island Analysis

**Figure 6:**
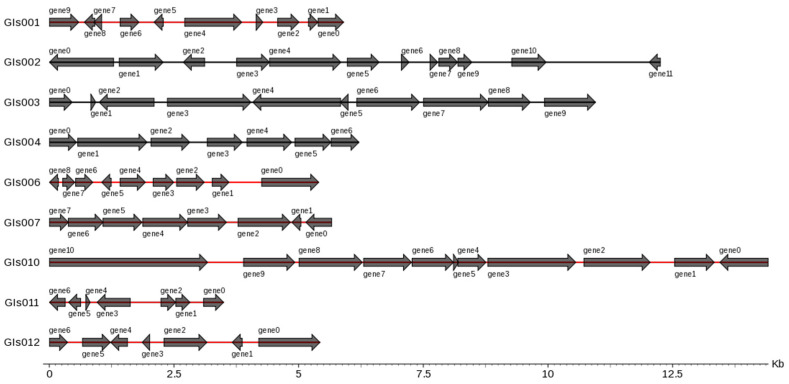
Gene distribution in gene islands of *B. cereus* SCL10 strain. On the left is the gene island ID, and on the right is the number and size of genes contained in the gene island. Horizontal coordinates are length scales. GIs shown in figure are less than 15 kb.

There were 12 genomic islands (GIs) in the whole genome of the *B. cereus* SCL10 strain, which were related to a variety of biological functions, such as pathogenic and adaptive mechanisms (Figure 6). Genes related to the flagellum included the flagellar biosynthesis regulator *flhF*; flagellar biosynthesis proteins *flhA, flhB, fliR,* and *fliP*; flagellar export apparatus protein *fliQ*; and flagellar motor switch protein *fliM*. Additionally, other metabolic regulatory factors were concluded in GIs004, such as the GTP-sensing transcriptional pleiotropic repressor *codY*, and were included in GIs009, such as phage integrase family protein, FAD-dependent oxidoreductase, alkyl hydro peroxidase AhpD family core domain-containing protein, spore photoproduct lyase, and deoxyribodipyrimidine photolyase (Table S8).

### 3.5. B. cereus SCL10 Strain Multiple Pathogenic Factors and Antibiotic Resistance

**Figure 7:**
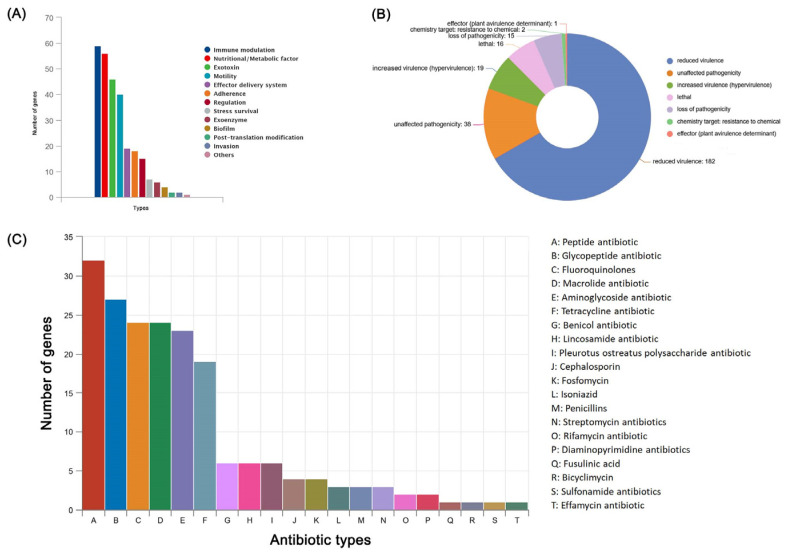
Pathogenic factors and antibiotic resistance analysis of *B. cereus* SCL10 strain. (**A**) Distribution of virulence factors. (**B**) Distribution of pathogen–host interaction genes. (**C**) Distribution of main top 20 antibiotic resistance genes.

Genes associated with virulence and stress response were annotated in the *B. cereus* SCL10 strain genome. The genome harbored 260 virulence genes aligned to the VFDB database. All virulence factors were categorized into 13 types (Figure 7A). The top four types, which included immune modulation, nutritional/metabolic factor, exotoxin, and motility, had different numbers of unigenes (Figure S4). In the pathogen–host interactions, the number of PHI-related genes of the *B. cereus* SCL10 strain was 273, mainly distributed in the categories of reduced virulence (66.7%) and unaffected pathogenicity (13.9%) (Figure 7B). In addition, the *B. cereus* SCL10 strain carried 192 antibiotic resistance genes of 20 major types (Figure 7C, Table S9). The top five types containing the largest numbers of genes were as follows: 32 peptide antibiotic genes, 27 glycopeptide antibiotic resistance genes, 24 fluoroquinolone genes, 24 macrolide resistance genes, and 23 aminoglycoside resistance genes. A small number of genes that mediated resistance to antibiotics, such as diaminopyrimidines, sulfa, and streptomycin, were also found in the genome.

### 3.6. Comparative Genomics of B. cereus SCL10 Strain

**Figure 8:**
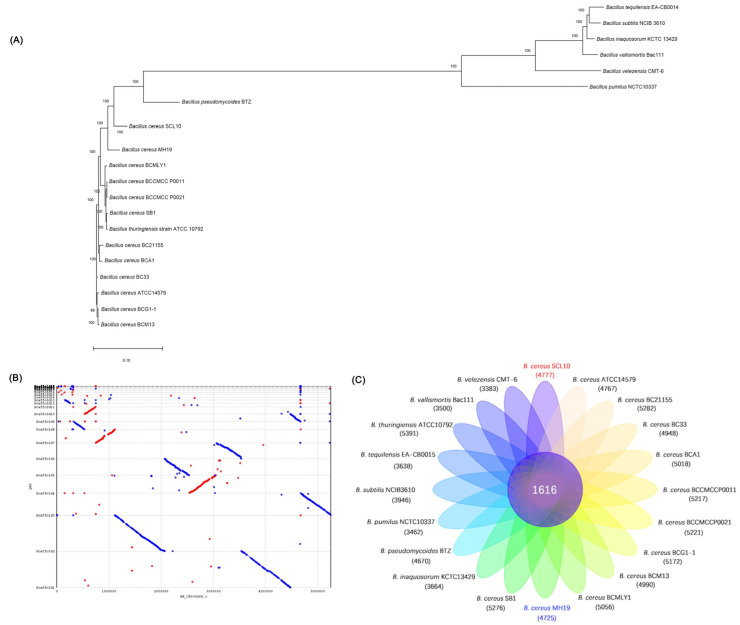
Comparative genomic and evolutionary analysis of *B. cereus* SCL10 strain and other 19 strains. (**A**) Phylogenetic analysis based on single-copy genes of 20 strains. (**B**) Synteny analysis of *B. cereus* SCL10 strain and *B. cereus* MH19 strain. Red represents forward alignment, blue represents reverse alignment, and gaps represent possible rearrangements between two genomes. (**C**) Venn diagram of gene family analysis showing shared and unique genes of 20 strains.

In addition to the general functional annotation of the *B. cereus* SCL10 strain, comparative genomics was also carried out to analyze its specific functions in comparison with those of other *Bacillus* genera. The phylogenetic tree was constructed based on the alignment of 1355 single copies of homologous genes from 20 strains (Figure 8A, Table S1). The *B. cereus* SCL10 strain, *B. cereus* MH19 strain, and other *Bacillus* sp. strains were located on the same large branch. In addition, the *B. cereus* SCL10 strain and *B. cereus* MH19 strain were located on adjacent branches and clustered closely to each other, with a confidence level of 100. Based on the phylogenetic tree, the synteny analysis of the *B. cereus* SCL10 strain and *B. cereus* MH19 strain was performed to explore their evolutionary relationship (Figure 8B). The genome sequences of the two strains had high similarity and a large number of homologous regions. At the same time, there was also a small amount of translocation and gene deletion. The blank area showed the area not aligned, indicating that the strain had unique genomic regions. Additionally, the core genome analysis was conducted by comparing the *B. cereus* SCL10 strain genome and the genomes of 19 other strains. The results revealed that the number of shared orthologous genes was 1616 among the 20 strains, and the *B. cereus* SCL10 strain and *B. cereus* MH19 strain showed 4777 and 4725 genes, respectively, with the smallest difference between them (Figure 8C). A further analysis revealed that 3853 unigenes were homologous, 770 unigenes were unique to the *B. cereus* MH19 strain, and 920 unigenes were unique to the *B. cereus* SCL10 strain.

### 3.7. Stress-Resistance Genes Related to Spores and Biofilms

In the *B. cereus* SCL10 strain genome, annotated stress-resistance genes were relevant to the general stress response and the main two aspects, spores and biofilms. From an overall perspective, five general stress response protein genes, forty-four spore-related genes, and five biofilm-related genes were identified (Table 2). These functional genes were annotated by comparison with the Swiss-Prot database. Comparison with the NR database also revealed a large number of spore-associated genes, whose identity was mostly 100. In addition to the stress response protein genes *yhaX*, *yugI*, *yceD*, *yceC*, and *SH*1215 annotated against the Swiss-Prot database, the universal stress protein-encoding gene *uspA* was also annotated against the NR database. Genes related to spores included small, acid-soluble spore protein (SASP), spore germination protein, different stage sporulation protein, spore maturation protein, septation protein, and sporulation transcription regulator, including *spo0/II/III/IV/V*, *gerE/I/L/P, sspF/H1/H2/I/K/O/N*, and so on. The sporulation membrane protein *ytaF*, membrane protein insertion efficiency factor *yidD*, and mutator protein *mutT* are worth noting (Table S10).

### 3.8. Stress-Resistance Genes Related to Other Biological Processes

In addition to the genes mentioned above, genes related to several other aspects of stress resistance, such as a cellular process, transporter, cold/salt resistance, oxidative stress resistance, transcriptional regulation, and DNA protection, were identified (Table 3). These genes, gene families, and superfamilies were derived from different databases, namely, Swiss-Prot, TCDB, Pfam, and GIs. Additional descriptions of these genes are provided in the Supplementary Materials (Table S11). Additionally, the NR database annotated (redox-sensitive) transcriptional regulation, cold tolerance, and DNA protection-related genes (Table S10). The cold/salt resistance genes were associated with cold shock proteins (*cspB, cspC, cspE*) and the (salt or low-temperature) stress-induced hydrophobic peptide (SHP) family. About transcriptional regulation, the genes transcriptional repressor *nrdR*, transcription antitermination protein *nusB*, redox-sensitive transcriptional repressor *rex,* and nutrient-sensitive regulator *codY* were annotated. For the DNA protection-related functions, a large number of genes were found, including DNA repair protein *recFOR* and *radA*, DNA protection during starvation proteins *dps1* and *dps2*, UV DNA damage endonuclease *uvsE*, DNA breaking–rejoining enzyme superfamily, spore photoproduct lyase, and deoxyribodipyrimidine photolyase. Moreover, there were special genes associated with inhibitors of programmed cell death *bcl-2* and the chloroplast envelope protein translocase (CEPT or Tic-Toc) family in the genome.

## 4. Discussion

Foodborne illnesses caused by pathogenic *B. cereus* occur worldwide, and this species poses a great challenge to the food and public health industries as a result of its extraordinary resistance, making it difficult to eradicate [24]. Present-day sterilization and detection techniques have improved the control of infection by this bacterium. Genomic studies of new isolates can uncover gene functions, explain biological mechanisms, and identify stress-resistance genes for better sterilization [25]. The *B. cereus* SCL10 strain, which remained active, was isolated from Co-60-inactivated watery sea cucumber (*A. molpadioides*) cryopreserved for more than 10 years. The survival of the SCL10 strain under radiation sterilization could be associated with predicted stress-resistance genes. Traditional experimental and identification methods have difficulty in fully analyzing the resistance of *B. cereus* and cannot uncover all of the stress-resistance genes. In this study, the whole genome of the *B. cereus* SCL10 strain was sequenced to investigate genes involved in defense response and resistance to adverse environments by WGS and a bioinformatics analysis.

Oxidative stress is an inherent defense response of organisms. Due to genetic history, oxidative damage is unavoidable in adverse environments, and organisms have evolved their metabolic systems to protect against oxidation [26]. Alkyl hydroperoxide reductase AhpC and its corresponding reducing systems (e.g., AhpF) were the first systems used by bacteria to decompose ROS [27]. The organic hydroperoxide reductase/osmotically induced bacterial protein C (Ohr/OsmC) family can defend against the attack of fatty acid hydroperoxides and peroxynitrite [28]. DNA-binding proteins from starved cells (Dps) act as oxidative DNA damage protectants [29]. The genes *dps1* and *dps2* were identified in the genome in this study. Dps performs dual actions to protect DNA by physical binding and reducing oxidative damage initiated by excess iron [30].

The significant induction of general stress proteins is one of the most notable stress responses of the bacterium [31]. Orthologous genes related to general stress protein (WP_001998456.1) were annotated in 31 GO functional modes, indicating a high number of annotations. Genes linked with stress response proteins such as *yhaX, yugI, yceD, yceC,* and SH1215 were present in the genome. Moreover, the ATP-dependent chaperone gene *clpB* (WP_000365408.1) was annotated in 32 GO modes, and *clpA* (NP_842649) and *clpP* (YP_007424642) were found in COG annotation. Unfolded proteins may be a common outcome of coping with different stresses. The chaperone Clp represents a key cellular component in bacterial survival and adaptation since it maintains cellular proteostasis to prevent protein misfolding [32]. Among the Clp chaperones, ClpB performs vital functions for protection in radiation environments through changes in ROS, antibiotics, and bactericidal molecules [33].

Spores, one of the special structures of *Bacillus* spp., can withstand adverse environments and make later virulence release possible [34]. Biofilms increase the resistance of spores or vegetative cells, which can protect *B. cereus* to different degrees [35]. In total, 49 low-redundancy protein-encoding genes were obtained by Swiss-Prot database annotation to determine exactly which genes regulate spore and biofilm formation. For spore formation, different stage sporulation proteins affect the corresponding stage, which can produce functionally important amino acids to construct the spore and maintain the capacity to survive and the potential for reactivation [36]. *SpoVG* and *Spo*0*A* are important in regulating sporulation and biofilm formation [37]. The spore germination protein GerP in the genome plays a major role in the coat (inner coat and outer coat) [38]. Spore maturation protein genes and *spoII/III/IV/V* were highly abundantly expressed during sporulation. The cold shock proteins CspB, CspC, and CspE also act in growth, biofilm formation during temperature decrease, and antibiotic resistance [39]. Prior studies showed that Clp also plays an important role in biofilm formation and bacterium-hesive material interactions in addition to general stress regulation [40].

As discussed above, the outer spore layer and its low-water-content core have a barrier effect under low temperatures and radiation. Moreover, DNA protection mechanisms are also available to prevent further damage to the spore [41]. Upon radiation exposure, the inner DNA is damaged; as a result, the number of DNA protection genes exhibits a wide distribution. The two major spore DNA repair pathways of *Bacillus subtilis* were also found in *B. cereus*, namely, nucleotide excision repair and spore photoproduct lyase, which are vital to resistance and DNA protection [42]. This may explain why *B. cereus* can remain active in food sterilized by radiation. Ultraviolet (UV) DNA damage endonuclease *uvsE* and DNA damage scanning *disA* in the genome showed that DNA was damaged. Thus, DNA protection and repair in spores can play an important role under radiation pressure. The DNA repair proteins RecA and RecFOR participate in synthesis-dependent strand annealing (SDSA) and recombination repair, during which the RecA-dependent accessory protein RadD greatly accelerates DNA strand exchange [43,44]. However, the mechanism of RecF remains to be further studied. In addition, 53 unigenes in metabolic pathways for replication and repair were annotated among the KEGG pathways. During the protection process, small, acid-soluble spore protein (SASP), an exclusive constituent of the spore core, acts as a major protectant of spore DNA since they saturate the spore DNA to alter the structures and properties dramatically [45]. Regarding photochemistry protection, UV-induced spore photoproducts (SPs) are repaired by spore photoproduct lyase (SPL), regarded as a major DNA repair enzyme in spore-forming genera [46].

## 5. Conclusions

This study provides a general description of the resistance of the *B. cereus* SCL10 strain at the gene level by WGS. The whole genome consisted of 34 scaffolds of 4,979,182 bp and 5167 coding sequences. By comparison with the GO, COG, KEGG, NR, and Swiss-Prot databases, various functional genes were annotated. The *B. cereus* SCL10 strain has evolved adaptive radiation-stress genes to survive under conditions of radiation with Co-60. In the annotation of stress-resistance genes, antioxidant genes included *ahpC*, *ahpF*, and *dps*. Spore- and biofilm-related genes included *spoVG*, *spo*0*A*, *gerP*, *cspC*, *cspE*, and *clpB*. DNA protection components included photolyase, SASP, and the genes *recA* and *radD*. In conclusion, a variety of genetic characteristics, especially oxidative stress-related genes, sporulation genes, biofilm-related genes, and DNA protection regulation genes, are likely to be responsible for the radiation-adapted phenotype of the *B. cereus* SCL10 strain. DNA protection is the most important factor in radiation resilience and mainly depends on nucleotide excision repair and the spore photoproduct lyase. We hope that our analysis of stress resistance and the underlying mechanism will provide important insight for protection against *B. cereus* infection and in turn contribute to food safety and public health.

## Figures and Tables

**Table 1:** Genome prediction statistics of *B. cereus* SCL10 strain.

Type	Length (bp)	Amount
Contig count	4,979,162	34
Genome size	4,979,162	
Gene average	807	
Contig N50	408,461	
GC content (%)	35.42	
Total length of coding sequence	4,167,474	5167
Protein-encoding genes with functional assignment		4955
Long terminal repeat	8525	115
Total transposon length	2061	33
Long scattered sequence	1853	25
Short scattered sequence	787	10
Rolling circle	0	0
Genomic island	137,391	12
Prophage	93,260	3
CRISPR	1805	6
Plasmid	0	0
Number of tRNAs	6850	89
Number of rRNA	5787	14
Number of sRNAs	542	6

**Table 2:** Genes related to general stress resistance of *B. cereus* SCL10 strain.

Function	Subject ID	Genes	Description	Number
general stress	O07539	*yhaX*	Stress response protein YhaX	
	P80870	*yugI*	General stress protein 13	
	P80875	*yceD*	General stress protein 16U	5
	P81100	*yceC*	Stress response protein SCP2	
	Q4L751	*SH*1215	Putative universal stress protein SH1215	
spores	A0RKU0	*whiA*	Putative sporulation transcription regulator	
	A7GJW0	*spoVG*	Putative septation protein SpoVG	
	A9VNW3	*sspO*	Small, acid-soluble spore protein O	
	B7IJW2	*sspI*	Small, acid-soluble spore protein I	
	C0H450	*spoVAEB*	Stage V sporulation protein AEB	
	C3P450	*sspN*	Small, acid-soluble spore protein N	
	C3PCY0	*sspK*	Small, acid-soluble spore protein K	
	C9RZ55	*splG*	Spore photoproduct lyase	
	O68685	*gerPC*	Probable spore germination protein GerPC	
	O68687	*gerPE*	Probable spore germination protein GerPE	
	O85467	*gerIA*	Spore germination protein GerIA	
	P04835	*SASP-C*5	Small, acid-soluble spore protein C5	
	P07373	*spoVE*	Stage V sporulation protein E	
	P07787	3 *SV*	Small, acid-soluble spore protein gamma type	
	P0A3T9	*gerPD*	Probable spore germination protein GerPD	
	P0A3V1	*sleB*	Spore cortex-lytic enzyme	
	P0A4F5	*sasP*-2	Small, acid-soluble spore protein 2	
	P0A4F7	*sspF*	Protein SspF	44
	P10572	*SASP-C*3	Small, acid-soluble spore protein C3	
	P11470	*gerE*	Spore germination protein GerE	
	P27643	*spoVK*	Stage V sporulation protein K	
	P35157	*spmA*	Spore maturation protein A	
	P35158	*spmB*	Spore maturation protein B	
	P37541	*yaaT*	Stage 0 sporulation protein YaaT	
	P37554	*spoVT*	Stage V sporulation protein T	
	P37558	*yabP*	Spore protein YabP	
	P37875	*spoVR*	Stage V sporulation protein R	
	P37956	*splB*	Spore photoproduct lyase	
	P40868	*spoVAC*	Stage V sporulation protein AC	
	P45693	*spoVS*	Stage V sporulation protein S	
	P49781	*spoIIIAD*	Stage III sporulation protein AD	
	P52928	*spo*0*A*	Stage 0 sporulation protein A	
	P62164	*gerPA*	Probable spore germination protein GerPA	
	P62186	*gerPF*	Probable spore germination protein GerPF	
	P62186	*gerPF*	Probable spore germination protein GerPF	
	Q00213	*SASP*-1	Small, acid-soluble spore protein 1	
	Q03524	*spoVD*	Stage V sporulation protein D	
	Q81AF2	*sspP*	Small, acid-soluble spore protein P	
	Q81I11	*sspH*1	Small, acid-soluble spore protein H 1	
	Q81QD6	*spoIISA*	Stage II sporulation protein SA	
	Q81SD1	*sspH*2	Small, acid-soluble spore protein H 2	
	Q81SW4	*spoIVA*	Stage IV sporulation protein A	
	Q93N68	*gerLC*	Spore germination protein GerLC	
	Q93N69	*gerLB*	Spore germination protein GerLB	
biofilms	O32233	*secG*	Probable protein-export membrane protein	
	P71021	*divIVA*	Septum site-determining protein DivIVA	
	Q01464	*minD*	Septum site-determining protein MinD	5
	Q72ZX1	*maf*	Septum formation protein Maf	
	Q732H0	*sepF*	Cell division protein SepF	

**Table 3:** Other genes related to stress resistance of *B. cereus* SCL10 strain.

Classification	Subject ID	Genes	Number	Source
Cellular process	A9VMG8	*gpsB*	1	Swiss-Prot
	2.A.6	RND Superfamily	2	TCDB
	CL0551.1	*bcl*-2	18	Pfam
Transporter	1.A.22	MscL Family	2	TCDB
	1.A.34	GJ-CC Family	2	
	2.A.41	CNT Family	7	
	2.A.88	VUT/ECF Family	2	
	2.A.9	Oxa1 Family	2	
Cold/salt resistance	P62169	*cspC*	1	Swiss-Prot
	Q45097	*cspB*	1	
	Q81QK2	*cspE*	1	
	9.B.12	SHP Family	1	TCDB
Oxidative stress resistance	O34762	*ohrA*	1	Swiss-Prot
	O34777	*ohrR*	1	
	Q81TR6	*spx* 1	1	
	Q9K813	*tpx*	1	
	WP_026594348.1	Thiol Reductase Thioredoxin	1	GIS011
Transcriptional regulation	A9VQG4	*rex*	1	Swiss-Prot
	A9VT66	*codY*	1	
	B7IXH3	*nusB*	1	
	C3P4Y3	*hfq*	1	
	C3PAG9	*nrdR*	1	
	WP_000421293.1	*codY*	1	GIS004
DNA protection	A0R883	*recF*	1	Swiss-Prot
	A0RH75	*lexA*	1	
	A9VHS5	*recO*	1	
	C1ER74	*noc*	1	
	C3LFM1	*uvsE*	1	
	C3P9M9	*disA*	1	
	P37572	*radA*	1	
	Q8RPQ1	*dps*1	1	
	Q8RPQ2	*dps*2	1	
	3.A.11	DNA-T Family	4	TCDB
	3.A.12	S-DNA-T Family	2	
	CL0382.3	DNA Breaking–Rejoining Enzyme Superfamily	10	Pfam
	WP_001039637.1	Spore Photoproduct Lyase	1	GIS009
	WP_001169829.1	Deoxyribodipyrimidine Photolyase	1	GIS009

## Data Availability

The datasets presented in this study can be found in online repositories. The names of the repository/repositories and accession number(s) can be found at: https://www.ncbi.nlm.nih.gov/, PRJNA1051368. The genome was accessed on 26 December 2023.

## Supplementary Materials

The following supporting information can be downloaded at: https://www.mdpi.com/article/10.3390/microorganisms12061168/s1, Figure S1: The colony morphology of the *B. cereus* SCL10 strain; Figure S2: Data validity analysis of whole genome of *B. cereus* SCL10 strain; Figure S3: Gene clusters and CAZy annotation of *B. cereus* SCL10 strain; Figure S4: Distribution of *B. cereus* SCL10 strain virulence factors; Table S1: Identification of 20 strains according to the phylogenetic tree; Table S2: Gene analysis for KEGG annotation of *B. cereus* SCL10 strain; Table S3: Gene analysis for COG annotation of *B. cereus* SCL10 strain; Table S4: Gene analysis for GO annotation of *B. cereus* SCL10 strain; Table S5: Gene analysis for Pfam annotation of *B. cereus* SCL10 strain; Table S6: Gene analysis for Swiss-Prot annotation of *B. cereus* SCL10 strain; Table S7: Gene analysis for TCDB annotation of *B. cereus* SCL10 strain; Table S8: Gene analysis for GI annotation of *B. cereus* SCL10 strain; Table S9: Gene analysis for the main antibiotic resistance of *B. cereus* SCL10 strain; Table S10: Gene analysis for various functions and stress resistance of B. cereus SCL10 in NR annotation; Table S11: Gene analysis for various stress resistance of *B. cereus* SCL10 in classification.
