# Supplementary material for: Functional Analysis of Stress Resistance of Bacillus cereus SCL10 Strain Based on Whole-Genome Sequencing
Source: Microorganisms. 2024 Jun 8;12(6):1168. doi: 10.3390/microorganisms12061168 (PMC11206075; doi:10.3390/microorganisms12061168)
Supplement: Supplementary file 1 [file microorganisms-12-01168-s001.zip › microorganisms-2971981-supplementary.pdf]

Functional Analysis of Stress Resistance of *Bacillus cereus* SCL10 Strain Based on Whole-Genome Sequencing

Yanzhen Mao [1,2], Ye Yang [1,2], Fu Lin [1,2], Hanyu Chu [1,2], Lijie Zhou [1,2], Jiaojiao Han [1,2], Jun Zhou [1,2,*] and Xiurong Su [1,2]

1 State Key Laboratory for Managing Biotic and Chemical Threats to the Quality and Safety of Agro-Products, Ningbo University, Ningbo 315832, China; mao2010111@163.com (Y.M.); 17815628500@163.com (Y.Y.); 18858047487@163.com (F.L.); 13462005107@163.com (H.C.); zhoulijie1113@163.com (L.Z.); hanjiaojiao@nbu.edu.cn (J.H.); suxiurong@nbu.edu.cn (X.S.)

2 School of Marine Science, Ningbo University, Ningbo 315832, China

* Correspondence: zhoujun1@nbu.edu.cn; Tel.: +86-574-87608368

Table S1. Identification of 20 strains according to the phylogenetic tree.

Number	Strains	Group
1	*B. cereus* BC33	group1
2	*B. cereus* ATCC14579	group2
3	*B. cereus* BCG1-1	group2
4	*B. cereus* BCM13	group2
5	*B. cereus* BC21155	group3
6	*B. cereus* BCA1	group3
7	*B. cereus* BCMLY1	group4
8	*B. cereus* BCCMCC P0011	group4
9	*B. cereus* BCCMCC P0021	group4
10	*B. cereus* SB1	group4
11	*B. thuringiensis* strain ATCC 10792	group4
12	***B. cereus* MH19**	group5
13	***B. cereus* SCL10**	group5
14	*B. pseudomycoides* BTZ	group5
15	*B. pumilus* NCTC10337	group5
16	*B. velezensis* CMT-6	group5
17	*B. vallismortis* Bac111	group5
18	*B. inaquosorum* KCTC 13429	group5
19	*B. tequilensis* EA-CB0015	group5
20	*B. subtilis* strain NCIB 3610	group5

Table S2. Genes analysis for KEGG annotation of *B. cereus* SCL10.

Map number	Map name	Gene number
**map02010**	ABC transporters	140
**map02020**	Two-component system	96
**map02024**	Quorum sensing	88
**map00230**	Purine metabolism	72
**map00620**	Pyruvate metabolism	55
**map00240**	Pyrimidine metabolism	53
**map00270**	Cysteine and methionine metabolism	52
**map03010**	Ribosome	52
**map00010**	Glycolysis / Gluconeogenesis	49
**map00190**	Oxidative phosphorylation	47
**map00260**	Glycine, serine and threonine metabolism	41
**map00330**	Arginine and proline metabolism	36
**map00550**	Peptidoglycan biosynthesis	34
**map00640**	Propanoate metabolism	34
**map00970**	Aminoacyl-tRNA biosynthesis	34
**map00500**	Starch and sucrose metabolism	33
**map02040**	Flagellar assembly	30
**map02030**	Bacterial chemotaxis	24
**map00730**	Thiamine metabolism	21
**map03060**	Protein export	21
**map03430**	Mismatch repair	21
**map03440**	Homologous recombination	21
**map01501**	beta-Lactam resistance	18
**map04112**	Cell cycle - Caulobacter	15
**map04122**	Sulfur relay system	15
**map00710**	Carbon fixation in photosynthetic organisms	14
**map03070**	Bacterial secretion system	14
**map03410**	Base excision repair	13
**map00920**	Sulfur metabolism	12
**map04146**	Peroxisome	11
**map03420**	Nucleotide excision repair	10
**map00195**	Photosynthesis	8

Table S3. Genes analysis for COG annotation of *B. cereus* SCL10.

Functional class	Class description	Gene number
**R**	General function prediction only	379
**E**	Amino acid transport and metabolism	362
**K**	Transcription	346
**S**	Function unknown	299
**J**	Translation, ribosomal structure and biogenesis	288
**G**	Carbohydrate transport and metabolism	252
**M**	Cell wall/membrane/envelope biogenesis	226
**P**	Inorganic ion transport and metabolism	210
**T**	Signal transduction mechanisms	209
**H**	Coenzyme transport and metabolism	202
**C**	Energy production and conversion	193
**I**	Lipid transport and metabolism	162
**O**	Posttranslational modification, protein turnover, chaperones	161
**L**	Replication, recombination and repair	145
**F**	Nucleotide transport and metabolism	128
**V**	Defense mechanisms	112
**Q**	Secondary metabolites biosynthesis, transport and catabolism	98
**D**	Cell cycle control, cell division, chromosome partitioning	70
**N**	Cell motility	58
**X**	Mobilome: prophages, transposons	56
**U**	Intracellular trafficking, secretion, and vesicular transport	39
**W**	Extracellular structures	4
**Z**	Cytoskeleton	3
**A**	RNA processing and modification	2
**B**	Chromatin structure and dynamics	1

Table S4. Genes analysis for GO annotation of *B. cereus* SCL10.

Subject ID	GO number	Subject description
WP_028397111.1	45	hypothetical protein [*Bacillus* sp. FJAT-14578]
WP_001990497.1	38	MULTISPECIES: hypothetical protein [*Bacillus cereus* group]
WP_053565554.1	34	hypothetical protein ACN91_27630 (plasmid) [*Bacillus cereus*]
WP_070173905.1	33	methyl-accepting chemotaxis protein McpC [*Bacillus thuringiensis*]
WP_000365408.1	32	ATP-dependent chaperone ClpB [*Bacillus cereus*]
WP_001182500.1	31	heme ABC transporter ATP-binding protein [*Bacillus cereus*]
WP_001998456.1	31	MULTISPECIES: general stress protein [*Bacillus*|*Bacillus anthracis*|*Bacillus cereus*|*Bacillus* sp.|*Bacillus thuringiensis*|*Streptococcus pneumoniae*]
EEK70243.1	29	ATPase [*Bacillus cereus* AH621]
WP_033663067.1	28	cobalt ABC transporter ATP-binding protein [*Bacillus cereus*]
WP_001033473.1	24	topology modulation protein [*Bacillus cereus*]
WP_025149637.1	24	MULTISPECIES: ATP-dependent Clp protease ATP-binding subunit ClpC [*Bacillus*]
WP_002116745.1	23	Uncharacterized protein BWINRASL_00954 [*Bacillus weihenstephanensis*]
EEK72625.1	23	Surface protein, LPXTG-motif cell wall anchor domain protein [*Bacillus cereus* AH621]
WP_000360715.1	21	pyrroline-5-carboxylate reductase [*Bacillus cereus*]
WP_000037046.1	21	NAD-dependent epimerase/dehydratase family protein [*Bacillus thuringiensis* MC28]
WP_000276563.1	21	MerR family transcriptional regulator [*Bacillus cereus*]
WP_002121674.1	20	nucleotide sugar dehydrogenase [*Bacillus cereus* BAG6X1-2]
WP_065845780.1	20	MULTISPECIES: bacteriocin ABC transporter ATP-binding protein [*Bacillus cereus* group]

WP_001131609.1	20	MULTISPECIES: septum formation initiator [*Bacillus cereus*|*Bacillus* mycoides|*Bacillus weihenstephanensis*|*Bacillus wiedmannii*]
WP_001219744.1	19	MULTISPECIES: alanine dehydrogenase [*Bacillus*|*Bacillus bombysepticus*|*Bacillus cereus*|*Bacillus* sp.|*Bacillus thuringiensis*|*Streptococcus pneumoniae*]
WP_033685340.1	19	polysaccharide biosynthesis protein EpsC [*Bacillus cereus*]
ABY45476.1	19	ATP-dependent Clp protease, ATP-binding subunit ClpX [*Bacillus weihenstephanensis* KBAB4]
WP_002158678.1	18	MULTISPECIES: ketol-acid reductoisomerase [*Bacillus cereus*|*Bacillus weihenstephanensis*]

Table S5. Genes analysis for Pfam annotation of *B. cereus* SCL10.

Clan	Description	Number
**CL0023.33**	P-loop containing nucleoside triphosphate hydrolase superfamily	1641
**CL0063.24**	FAD/NAD(P)-binding Rossmann fold Superfamily	1175
**CL0123.17**	Helix-turn-helix clan	1001
**CL0257.8**	N-acetyltransferase like	369
**CL0036.23**	Common phosphate binding-site TIM barrel superfamily	202
**CL0061.12**	PLP dependent aminotransferase superfamily	144
**CL0015.19**	Major Facilitator Superfamily	127
**CL0028.21**	Alpha/Beta hydrolase fold	118
**CL0025.13**	His Kinase A (phospho-acceptor) domain	105
**CL0062.12**	APC superfamily	97
**CL0344.3**	4Fe-4S ferredoxins	95
**CL0039.11**	HUP - HIGH-signature proteins, UspA, and PP-ATPase	83
**CL0020.24**	Tetratrico peptide repeat superfamily	64
**CL0184.10**	Drug/Metabolite transporter superfamily	52
**CL0105.12**	Barrel sandwich hybrid superfamily	33
**CL0198.15**	Helix-hairpin-helix superfamily	26
**CL0553.1**	Helical backbone metal receptor superfamily	19
**CL0551.1**	Bcl-2 inhibitors of programmed cell death	18
**CL0322.3**	RND permease superfamily	11
**CL0382.3**	DNA breaking-rejoining enzyme superfamily	10
**CL0575.1**	Translation proteins of Elongation Factors superfamily	10
**CL0318.3**	Cytochrome c superfamily	9
**CL0376.3**	Cytochrome oxidase biogenesis family	9
**CL0328.3**	Transmembrane di-heme cytochrome superfamily	8
**CL0591.1**	Transketolase C-terminal domain-like superfamily	6
**CL0524.2**	Antigen MPT63/MPB63 (immunoprotective extracellular	5
**CL0057.13**	MetJ/Arc repressor, antitoxin of TA systems superfamily	5
**CL0570.1**	Protease propeptides/inhibitors	5
**CL0204.10**	Bacterial adhesin superfamily	4
**CL0206.10**	Transcriptional repressor beta-barrel domain	4
**CL0324.3**	Homing endonuclease-like superfamily	3

Table S6. Genes analysis for Swiss-Prot annotation of *B. cereus* SCL10.

Subject ID	Protein ID	Swissprot Gene
**A0RKU0**	Putative sporulation transcription regulator WhiA	*whiA*
**A9VMG8**	Cell cycle protein GpsB	*gpsB*
**A9VQG4**	Redox-sensing transcriptional repressor Rex	*rex*
**B7HCU0**	Chaperone protein DnaK	*dnaK*
**B7IJP9**	Probable manganese-dependent inorganic pyrophosphatase	*ppaC**
**B7IJW2**	Small, acid-soluble spore protein I	*ssp*I*
**B7IST2**	Ribosomal RNA small subunit methyltransferase G	*rsmG**
**B7ITN1**	Protein RecA	*recA*
**B7ITN1**	Protein RecA	*recA**
**B7IUI3**	Ribosome-recycling factor	*frr**
**B7IXH3**	Transcription antitermination protein NusB	*nusB**
**C3P4Y3**	RNA-binding protein Hfq	*hfq*
**C3P625**	Transcription factor FapR	*fapR*
**C3P661**	Bifunctional protein PyrR	*pyrR*
**C3PAG9**	Transcriptional repressor NrdR	*nrdR*
**P62169**	Cold shock-like protein CspC	*cspC*
**P62172**	HTH-type transcriptional regulator GlnR	*glnR*
**Q732H0**	Cell division protein SepF	*sepF**
**Q732R1**	Ribosome-binding factor A	*rbfA*
**Q81L09**	S-adenosylmethionine decarboxylase proenzyme 1	*speH*1
**Q81QK2**	Cold shock-like protein CspE	*cspE*
**Q81SW4**	Stage IV sporulation protein A	*spo*IV*A*
**Q81TR6**	Regulatory protein Spx 1	*spxA*1
**Q8RPQ2**	DNA protection during starvation protein 2	*dps*2

Note, *Bacillus cereus*-specific proteins are marked with *.

Table S7. Genes analysis for TCDB annotation of *B. cereus* SCL10.

Transporter Class	Class Discription	Subclass Discription	Gene Number
**1**	Channels/Pores	Alpha-Type Channels	17
		Beta-Barrel Porins	1
		Pore-Forming Toxins (Proteins and Peptides)	14
		Holins	7
**2**	Electrochemical Potential-driven Transporters	Porters (uniporters, symporters, antiporters)	150
**3**	Primary Active Transporters	P-P-bond-hydrolysis-driven transporters	173
		Decarboxylation-driven transporters	1
		Oxidoreduction-driven transporters	25
**4**	Group Translocators	Phosphotransfer-driven Group Translocators	15
		Acyl CoA ligase-coupled transporters	1
		Polysaccharide Synthase/Exporters	1
**5**	Transmembrane Electron Carriers	Transmembrane 2-electron transfer carriers	1
**8**	Accessory Factors Involved in Transport	Auxiliary transport proteins	6
**9**	Incompletely Characterized Transport Systems	Recognized transporters of unknown biochemical mechanism	21
		Putative transport proteins	22

Table S8. Genes analysis for GIs annotation of *B. cereus* SCL10.

GIs	Gene ID	Identity	Subject ID	Subject description
**GIs001**	GM004836	95.9	WP_065845777.1	hypothetical protein [*Bacillus cereus*]
	GM004837	96.8	OFC88493.1	hypothetical protein [*Bacillus thuringiensis*, BTGOE4_59540]
	GM004838	93.7	WP_016113411.1	transcriptional regulator [*Bacillus cereus*]
	GM004839	95.5	EEL96321.1	Uncharacterized protein BWGO95_03055 [*Bacillus weihenstephanensis*]
	GM004840	49.1	AHA71801.1	collagen triple helix repeat-containing protein [*Bacillus thuringiensis* YBT-1518]
	GM004841	91.9	EOO75196.1	transposase, partial [*Bacillus cereus* BAG1O-1]
	GM004842	73.3	SCC54390.1	Uncharacterized protein BTT61001_04243 [*Bacillus thuringiensis*]
	GM004845	97.4	WP_000162758.1	hypothetical protein [*Bacillus cereus*]
**GIs002**	GM004894	87.3	WP_026590597.1	MFS transporter [*Bacillus* sp. UNC437CL72CviS29]
	GM004895	90.1	WP_026590596.1	hypothetical protein [*Bacillus* sp. UNC437CL72CviS29]
	GM004896	98.6	WP_048560210.1	transposon DNA-invertase [*Bacillus cereus*]
	GM004897	96.8	WP_062821062.1	DNA-binding response regulator [*Bacillus cereus*]
	GM004898	96.6	WP_062821061.1	Sensory transduction histidine kinase (with HAMP domain) [*Bacillus cereus*]
	GM004899	89.6	WP_061182347.1	hypothetical protein [*Bacillus cereus*]
	GM004901	96.1	EJQ37333.1	hypothetical protein [*Bacillus cereus* BAG5X1-1, IEE_05265]
	GM004902	88.7	WP_002107124.1	hypothetical protein [*Bacillus cereus*]
	GM004903	97.8	WP_000052523.1	hypothetical protein [*Bacillus cereus*]
	GM004904	98.7	WP_002107121.1	DNA-binding response regulator [*Bacillus cereus*]
**GIs003**	GM000640	96	WP_002122107.1	Uncharacterized protein BWINRASL_03859 [*Bacillus weihenstephanensis*]
	GM000642	98.4	WP_002122102.1	maltodextrin import ATP-binding protein MsmX [*Bacillus cereus*|*Bacillus weihenstephanensis*]
	GM000643	98	SCM96938.1	Oligo-1, 6-glucosidase [*Bacillus weihenstephanensis*]
	GM000644	93.7	WP_000471772.1	hypothetical protein [*Bacillus cereus*]

	GM000646	98.1	WP_000709932.1	cyclodextrin-binding protein [*Bacillus cereus*]
	GM000647	98.6	WP_001194981.1	arabinogalactan ABC transporter permease [*Bacillus cereus*]
	GM000648	98.9	WP_001022600.1	binding--dependent transport system inner membrane component family protein [*Bacillus thuringiensis*]
	GM000649	98.5	WP_048530777.1	LacI family transcriptional regulator [*Bacillus cereus*]
	GM000834	100	WP_000526274.1	ATP dependent peptidase CodWX, CodW component. Threonine peptidase. MEROPS family T01B [*Bacillus* sp.]
	GM000835	99.6	WP_000550075.1	ATP-dependent protease ATP-binding subunit HslU [*Bacillus cereus*]
	GM000836	100	WP_000421293.1	GTP-sensing transcriptional pleiotropic repressor CodY [*Bacillus cereus*|*Bacillus weihenstephanensis*]
**GIs004**	GM000837	100	WP_000111485.1	MULTISPECIES: 30S ribosomal protein S2 [*Bacillus*|*Bacillus bombysepticus*|*Bacillus cereus*|*Bacillus* sp.]
	GM000838	100	EEL86594.1	Elongation factor Ts [*Bacillus cereus*]
	GM000839	100	WP_000042664.1	Uridylate kinase [*Bacillus cereus*]
	GM000840	100	WP_000531505.1	MULTISPECIES: ribosome-recycling factor [*Bacillus*|*Bacillus cereus*|*Bacillus thuringiensis*]
	GM001319	85	KFN03413.1	flagellar hook-basal body family protein [*Bacillus mycoides*]
	GM001320	98.6	WP_000446160.1	MULTISPECIES: flagellar biosynthesis regulator FlhF [*Bacillus cereus*|*Bacillus thuringiensis*]
	GM001321	98.5	CUB30759.1	Flagellar biosynthesis protein FlhA [*Bacillus cereus*]
	GM001322	99.1	WP_001040123.1	flagellar biosynthetic protein FlhB [*Bacillus cereus*]
	GM001323	98.4	WP_001055702.1	MULTISPECIES: flagellar biosynthesis protein FliR [*Bacillus*|*Bacillus* sp.]
	GM001324	100	WP_001098085.1	MULTISPECIES: flagellar export apparatus protein FliQ [*Bacillus*|*Bacillus anthracis*|*Bacillus cereus*|*Bacillus* sp.]
	GM001325	99.6	WP_001220581.1	flagellar biosynthesis protein FliP [*Bacillus cereus*]
**GIs005**	GM001326	100	WP_000121171.1	hypothetical protein [*Bacillus cereus*]
	GM001327	97.5	WP_002011996.1	MULTISPECIES: flagellar motor switch protein FliN [*Bacillus cereus*|*Bacillus mycoides*|*Bacillus weihenstephanensis*]
	GM001328	98.5	WP_000012185.1	flagellar motor switch protein FliM [*Bacillus cereus*]
	GM001329	100	EEK51343.1	Flagellar motor switch protein [*Bacillus cereus*|*Streptococcus pneumoniae*]
	GM001330	93.9	WP_000635241.1	MULTISPECIES: lytic transglycosylase [*Bacillus anthracis*|*Bacillus cereus*]
	GM001331	89.3	ABV23538.1	flagellin A [*Bacillus thuringiensis serovar alesti*]
	GM001332	94	WP_061883965.1	flagellin [*Bacillus thuringiensis*|*Streptococcus pneumoniae*]

	GM001333	94.9	ABV23563.1	flagellin B2 [*Bacillus thuringiensis*]
	GM001335	90.3	WP_000274747.1	hypothetical protein [*Bacillus cereus*]
	GM001336	99	WP_064476536.1	chemotaxis protein CheV [*Bacillus wiedmannii*]
	GM001337	94.4	WP_000068319.1	Uncharacterized protein BC141101_00964 [*Bacillus cereus*]
	GM000446	97.7	WP_061401533.1	hypothetical protein [*Bacillus thuringiensis*]
	GM000447	91.7	EAO54304.1	hypothetical protein [*Bacillus thuringiensis*]
	GM000448	98.6	WP_070144825.1	type II 3-dehydroquinate dehydratase [*Bacillus weihenstephanensis*]
	GM000449	99.2	WP_002173421.1	MULTISPECIES: peptidase M24 [*Bacillus*]
**GIs006**	GM000450	100	WP_016080316.1	MULTISPECIES: elongation factor P [*Bacillus*|*Bacillus cereus*|*Bacillus* sp.|*Bacillus thuringiensis*]
	GM000451	93.4	WP_000866175.1	hypothetical protein [*Bacillus cereus*]
	GM000452	88	WP_048374160.1	GNAT family acetyltransferase [*Bacillus* sp. LK2]
	GM000453	95.7	WP_001991444.1	conserved hypothetical protein [*Bacillus anthracis*|*Bacillus cereus*| |*Bacillus subtilis*|*Bacillus thuringiensis*]
	GM000454	98.4	WP_000665901.1	stage III sporulation protein AA [*Bacillus cereus*]
	GM001645	99.4	EEM29740.1	Leucine-responsive regulatory protein [*Bacillus thuringiensis*]
	GM001646	98.3	WP_025150950.1	MULTISPECIES: DUF3913 domain-containing protein [*Bacillus*]
	GM001647	94.1	WP_001198824.1	AbrB family transcriptional regulator [*Bacillus cereus*]
	GM001648	89.9	WP_025150952.1	ABC transporter permease [*Bacillus* sp. H1a]
**GIs007**	GM001649	96.3	WP_061131203.1	ABC transporter ATP-binding protein [*Bacillus cereus*]
	GM001650	93.8	WP_001086851.1	hypothetical protein [*Bacillus cereus*]
	GM001651	94.4	SCB67156.1	ABC transporter ATP-binding protein [*Bacillus weihenstephanensis*]
	GM001652	96.8	CUB12421.1	HTH-type transcriptional repressor YtrA [*Bacillus cereus*]
	GM002413	95.6	WP_044810492.1	integrase [*Bacillus cereus*]
	GM002414	96.8	WP_016122140.1	hypothetical protein [*Bacillus cereus*]
**GIs008**	GM002415	100	WP_044810494.1	hypothetical protein [*Bacillus cereus*]
	GM002416	94.7	WP_044810495.1	hypothetical protein [*Bacillus cereus*]

	GM002417	47.8	WP_016205419.1	hypothetical protein [*Bacillus nealsonii*]
	GM002418	96.7	WP_042514689.1	hypothetical protein [*Bacillus cereus*, BG03_952]
	GM002419	91.8	WP_025147644.1	hypothetical protein [*Bacillus* sp. H1a]
	GM002421	82.5	WP_053565554.1	hypothetical protein ACN91_27630 (plasmid) [*Bacillus cereus*]
	GM002422	84	WP_000013830.1	transcriptional regulator [*Bacillus cereus*]
	GM002423	92	WP_044783822.1	hypothetical protein [*Bacillus thuringiensis*]
	GM002424	87.1	WP_070180723.1	hypothetical protein [*Bacillus thuringiensis*]
	GM002425	91.7	WP_065223786.1	hypothetical protein [*Bacillus cereus*]
	GM002426	87.1	WP_000580589.1	transcriptional regulator [*Bacillus cereus*]
	GM002427	72.3	WP_017474986.1	TaqI-like C-terminal specificity domain-containing protein [*Bacillus licheniformis*]
	GM002428	69.7	WP_026080877.1	MULTISPECIES: transcriptional regulator [*Bacillus licheniformis*|Bacteria]
	GM002429	74.3	WP_017474988.1	MULTISPECIES: hypothetical protein [*Bacillus licheniformis*,|Bacteria]
	GM002432	94.4	WP_070129026.1	hypothetical protein [*Bacillus weihenstephanensis*]
	GM002433	99.2	WP_002142229.1	MULTISPECIES: hypothetical protein [*Bacillus cereus*|*Bacillus thuringiensis*|*Bacillus weihenstephanensis*]
	GM002434	98.3	AOM13154.1	hypothetical protein [*Bacillus thuringiensis* Bt18247, BTI247_47840]
	GM002435	95.2	WP_001068199.1	hypothetical protein [*Bacillus cereus*]
	GM002869	99.8	SDD47479.1	hypothetical protein [*Bacillus cereus*, SAMN04487767_106176]
	GM002870	97.7	WP_048525285.1	hypothetical protein [*Bacillus cereus*]
	GM002871	100	SDD47369.1	Phage integrase family protein [*Bacillus cereus*]
	GM002872	99.6	SDD47328.1	hypothetical protein [*Bacillus cereus*, SAMN04487767_106172]
**GIs009**	GM002873	99.4	WP_001039637.1	MULTISPECIES: spore photoproduct lyase [*Bacillus*|*Bacillus* sp.|*Bacillus thuringiensis*]
	GM002874	98.9	WP_014895265.1	MULTISPECIES: transcriptional regulator SplA [*Bacillus*|*Bacillus* sp.|*Bacillus thuringiensis*]
	GM002875	100	WP_000114446.1	MULTISPECIES: hypothetical protein [*Bacillus*|*Bacillus cereus*|*Bacillus* sp.|*Bacillus thuringiensis*]
	GM002876	99.3	WP_014895264.1	MULTISPECIES: hypothetical protein [*Bacillus*|*Bacillus thuringiensis*]
	GM002878	99.7	WP_000684085.1	NAD dependent epimerase/dehydratase family protein [*Bacillus thuringiensis*]

	GM002879	100	WP_000063807.1	bacterial regulatory, arsR family protein [*Bacillus thuringiensis* HD1002]
	GM002880	100	WP_000849456.1	MULTISPECIES: hypothetical protein [*Bacillus*|*Bacillus* sp.|*Bacillus thuringiensis*]
	GM002881	98.2	EAL12162.1	hypothetical protein [*Bacillus cereus* G9241]
	GM002882	99.2	WP_000455786.1	alkylhydroperoxidase AhpD family core domain-containing protein [*Bacillus cereus* BAG2X1-3]
	GM002883	100	EEM28289.1	hypothetical protein [*Bacillus thuringiensis*]
	GM002884	97.7	WP_001169829.1	deoxyribodipyrimidine photo-lyase [*Bacillus cereus*]
	GM002885	97.9	WP_001093173.1	transcriptional regulator [*Bacillus cereus*]
	GM002886	99.2	WP_000225819.1	MFS family major facilitator transporter [*Bacillus thuringiensis* IBL 4222]
	GM002887	99	WP_065223165.1	MULTISPECIES: FAD-dependent oxidoreductase [*Bacillus cereus*|*Bacillus thuringiensis*]
	GM002888	96.3	WP_003310225.1	MULTISPECIES: hypothetical protein [*Bacillus*|*Bacillus thuringiensis*]
	GM002889	100	WP_001069278.1	hypothetical protein [*Bacillus cereus*]
	GM002890	97.6	WP_014895258.1	MULTISPECIES: hypothetical protein [*Bacillus*|*Bacillus* sp.|*Bacillus thuringiensis*|*Bacillus thuringiensis*,]
	GM002891	100	WP_014895257.1	MULTISPECIES: hypothetical protein [*Bacillus*|*Bacillus* sp.|*Bacillus thuringiensis*|*Bacillus thuringiensis*,]
	GM002892	100	WP_000738547.1	hypothetical protein A9L49_29545 (plasmid) [*Bacillus cereus*|*Bacillus* sp.| |*Bacillus weihenstephanensis*,|*Bacillus wiedmannii*]
	GM002893	100	WP_033658047.1	transcription factor YdeB [*Bacillus cereus*]
	GM002894	93.1	WP_000957102.1	Possible acyl-[acyl-carrier protein] desaturase [*Bacillus cereus*]
	GM002895	100	WP_000291468.1	MULTISPECIES: hypothetical protein [*Bacillus*|*Bacillus cereus*|*Bacillus thuringiensis*]
	GM002896	90.9	WP_044810125.1	hypothetical protein, partial [*Bacillus cereus*]
	GM002897	87.6	SEG86934.1	transposase [*Bacillus* sp. ok061]
	GM002898	68.5	EJS54996.1	hypothetical protein [*Bacillus cereus*]
	GM002899	87.2	SDD46521.1	hypothetical protein [*Bacillus cereus*, SAMN04487767_106151]
	GM002900	90.8	SDD46477.1	impB/mucB/samB family protein [*Bacillus cereus*]
	GM002901	96.7	SCN32977.1	Uncharacterized protein BC067498_03013 [*Bacillus cereus*]
**GIs010**	GM003030	92.7	WP_001003811.1	hypothetical protein [*Bacillus cereus*]
	GM003031	94.3	WP_016104920.1	glyoxalase/bleomycin resistance protein/dioxygenase superfamily protein [*Bacillus cereus*]

	GM003032	98.6	WP_002201076.1	alpha-glucosidase/alpha-galactosidase [*Bacillus cereus*]
	GM003033	98.1	SCC19615.1	Uncharacterized protein BW664_02005 [*Bacillus weihenstephanensis*]
	GM003034	98.9	WP_000561705.1	hypothetical protein [*Bacillus cereus*]
	GM003035	100	EJS63106.1	hypothetical protein [*Bacillus cereus* BAG2X1-1, ICU_04712]
	GM003036	98.5	WP_000629016.1	sugar ABC transporter permease [*Bacillus cereus*]
	GM003037	99.7	EJQ43397.1	hypothetical protein [*Bacillus cereus* BAG5X1-1, IEE_03575]
	GM003038	98.6	EJS68597.1	hypothetical protein [*Bacillus cereus* BAG2X1-3, ICY_04671]
	GM003039	98.8	WP_000258159.1	AraC family transcriptional regulator [*Bacillus cereus*]
	GM003040	93.8	WP_000867732.1	excinuclease ABC subunit A [*Bacillus cereus*]
	GM003246	95.4	WP_060488665.1	ABC transporter permease [*Bacillus cereus*]
	GM003247	92.2	WP_000500109.1	MULTISPECIES: alpha/beta hydrolase [*Bacillus*|*Bacillus* sp.]
**GIs011**	GM003248	92.9	WP_000969901.1	hypothetical protein [*Bacillus wiedmannii*]
	GM003249	89.6	WP_035430914.1	hypothetical protein [*Bacillus* sp. UNC322MFChir4.1]
	GM003251	93.7	WP_042983604.1	phage integrase family protein [*Bacillus* mycoides]
	GM003252	99	WP_026594348.1	thiol reductase thioredoxin [*Bacillus* sp.]
	GM004052	98	WP_000913728.1	cardiolipin synthetase [*Bacillus cereus*]
	GM004053	100	WP_002162205.1	hypothetical protein [*Bacillus cereus*]
	GM004054	95.1	WP_070146270.1	peptidylprolyl isomerase [*Bacillus weihenstephanensis*]
**GIs012**	GM004055	98	EEK80150.1	hypothetical protein [*Bacillus cereus* R309803, bcere0009_9110]
	GM004056	98.2	WP_029437755.1	MULTISPECIES: wall-associated protein [*Bacillus cereus*]
	GM004057	99.5	WP_000834922.1	HTH-type transcriptional regulator hpr [*Bacillus cereus*]
	GM004058	99.2	WP_002116745.1	Uncharacterized protein BWINRASL_00954 [*Bacillus weihenstephanensis*]

Table S9. Genes analysis for the main antibiotic resistance of *B. cereus* SCL10.

Types	Resistant genes	Number
**Peptide antibiotic**	*bcrA*, *bcrB*, *arnA*, *bacA*, *pmrE*, *mprF*, *pmrF*, *rosA*, *tsnr*, *vanRI*, *vanYB*	32
**Glycopeptide antibiotic**	*vanG*, *vanHO*, *vanL*, *vanRB*, *vanRE*, *vanRF*, *vanRG*, *vanRM*, *vanSB*, *vanSC*, *vanSF*, *vanSI*, *vanTG*, *vanWI*	27
**Fluoroquinolones**	*patA*, *mfd*, *patB*, *blt*, *arlR*, *arlS*	24
**Macrolide antibiotic**	*lmrP*, *efrA*, *macB*, *oleC*	24
**Aminoglycoside antibiotic**	*baeS*, *cpxA*, *sav*1866, *mdtC*, *novA*, *AAC(6')-34*, *ANT(6)-Ia*, *pmpM*, *AAC(3)-IIIa*	23
**Tetracycline antibiotic**	*hp1*184, *mepA*, *otr(A)*, *tcr*3, *tet(38)*, *tetA(48)*, *tetA(60)*, *tetB(48)*, *tetB(60)*	19
**Benicol antibiotic**	*cat*, *catB*2, *cmlv*, *cmr*, *mexN*	6
**Lincosamide antibiotic**	*lmrB*, *lmrD*	6
**Pleurotus ostreatus**		
**polysaccharide antibiotic**	*taeA*	6
**Cephalosporin**	*smeS*	4
**Fosfomycin**	*fosB*, *mdtG*, *murA*	4
**Isoniazid**	*fabI*	3
**Penicillins**	*bla*1, *mecC*	3
**Streptomycin antibiotics**	*msrB*, *vgaB*, *cfrA*	3
**Rifamycin antibiotic**	*rphB*	2
**Diaminopyrimidine antibiotics**	*dfrE*, *dfrK*	2
**Fusulinic acid**	*fusD*	1
**Bicyclimycin**	*bcr*-1	1
**Sulfonamide antibiotics**	*sul*3	1
**Effamycin antibiotic**	*EF-Tu* mutants	1

Table S10. Genes analysis for various functions and stress resistance of *B. cereus* SCL10 in NR annotation.

Subject ID	Identity	E value	Subject description
WP_000168890.1	100	1.10E-25	MULTISPECIES: acid-soluble spore protein [*Bacillus*|*Bacillus cereus*|*Bacillus* sp.|*Bacillus thuringiensis*|*Bacillus wiedmannii*]
WP_001066674.1	100	8.80E-77	MULTISPECIES: universal stress protein UspA [*Bacillus cereus*|*Bacillus weihenstephanensis*]
WP_002034279.1	99.5	4.10E-109	MULTISPECIES: sporulation membrane protein YtaF [*Bacillus cereus* group]
EEK48463.1	100	4.80E-30	Small, acid-soluble spore protein I [*Bacillus cereus*|*Bacillus thuringiensis*]
WP_000359781.1	100	3.20E-70	MULTISPECIES: organic hydroperoxide resistance protein [*Bacillus*|*Bacillus* anthracis|*Bacillus cereus*|*Bacillus* sp.|*Bacillus thuringiensis*|*Bacillus weihenstephanensis*|*Bacillus thuringiensis*]
WP_000350665.1	98.8	4.40E-201	spore coat protein YsxE [*Bacillus cereus*]
WP_000466737.1	100	5.20E-186	cell shape determining, MreB/Mrl family protein [*Bacillus* anthracis|*Bacillus cereus*|*Bacillus thuringiensis*]
WP_000704116.1	100	1.50E-70	transcriptional regulator, BadM/Rrf2 family [*Bacillus* anthracis|*Bacillus cereus*|*Bacillus* sp.|*Bacillus thuringiensis*|[*Bacillus thuringiensis*]]
WP_001221200.1	100	2.70E-69	Holliday junction DNA helicase [*Bacillus cereus*]
WP_000828145.1	100	9.90E-60	Membrane-attached cytochrome c550 [*Bacillus cereus*|*Bacillus* sp.|*Bacillus thuringiensis*|*Bacillus wiedmannii*]
WP_000665901.1	98.4	1.40E-169	stage III sporulation protein AA [*Bacillus cereus*]
WP_000020914.1	100	5.70E-28	Stage III sporulation protein AC/AD protein family protein [*Bacillus anthracis*|*Bacillus bombysepticus*|*Bacillus cereus*|*Bacillus gaemokensis*|*Bacillus mycoides*|*Bacillus pseudomycoides*|*Bacillus* sp.|*Bacillus subtilis*|*Bacillus thuringiensis*|*Bacillus toyonensis*|*Bacillus weihenstephanensis*|*Bacillus wiedmannii*|*Streptococcus pneumoniae*|*Bacillus thuringiensis*]
OFD73945.1	100	8.50E-71	stage V sporulation protein AB [*Bacillus weihenstephanensis*]
WP_065382565.1	100	1.30E-53	cytochrome c oxidase subunit IVB [*Bacillus cereus*]
WP_000421293.1	100	2.40E-135	GTP-sensing transcriptional pleiotropic repressor CodY [*Bacillus cereus*|*Bacillus weihenstephanensis*]
WP_062821667.1	99.7	9.50E-202	transcription termination/antitermination protein NusA [*Bacillus cereus*]
EAO51941.1	100	1.00E-37	Stage V sporulation protein S [*Bacillus thuringiensis* serovar israelensis ATCC 35646]
WP_001288800.1	100	3.90E-100	MULTISPECIES: spore coat protein E [*Bacillus*|*Bacillus* anthracis|*Bacillus cereus*|*Bacillus* sp.|*Bacillus thuringiensis*|*Bacillus wiedmannii*|*Streptococcus pneumoniae*|*Bacillus thuringiensis*]

EEL65383.1	100	2.90E-77	DNA protection during starvation protein [*Bacillus cereus*|*Bacillus thuringiensis*]
WP_002118476.1	100	2.70E-25	MULTISPECIES: sporulation protein YjcZ [*Bacillus cereus* group]
WP_000113539.1	100	5.20E-29	MULTISPECIES: small, acid-soluble spore protein, alpha/beta type [*Bacillus cereus*|*Bacillus* mycoides|*Bacillus* sp.|*Bacillus weihenstephanensis*]
WP_001019753.1	100	7.40E-24	Small, acid-soluble spore protein H [*Bacillus cereus*]
WP_000007806.1	100	8.60E-16	sporulation protein YjcZ [*Bacillus cereus*]
EAO55963.1	100	1.30E-29	Cold shock protein [*Bacillus thuringiensis* serovar israelensis ATCC 35646]
EEL06980.1	100	1.20E-54	Cell cycle protein gpsB [*Bacillus cereus*|*Bacillus mycoides*]
WP_002201161.1	100	2.80E-90	MULTISPECIES: spore maturation protein [*Bacillus cereus*|*Bacillus weihenstephanensis*]
WP_000241212.1	100	3.50E-24	small acid-soluble spore protein D (minor alpha/beta-type SASP) [*Bacillus* anthracis|*Bacillus cereus*|*Bacillus* mycoides|*Bacillus* sp.|*Bacillus thuringiensis*|*Bacillus weihenstephanensis*|*Bacillus wiedmannii*]
WP_000421885.1	100	4.70E-139	oxidoreductase, short chain dehydrogenase/reductase family superfamily [*Bacillus cereus* G9241]
WP_001003333.1	100	2.80E-23	competence protein ComZ [*Bacillus* sp. 112mf]
SCB95116.1	100	3.90E-26	Spore germination protein gerPD [*Bacillus cereus*]
WP_000301521.1	100	1.60E-30	cold-shock DNA-binding protein family [*Bacillus cereus*]
EJS74023.1	100	2.90E-33	stage III sporulation protein D [*Bacillus cereus*|*Bacillus thuringiensis*]
WP_000400858.1	100	6.70E-52	hxlR-like helix-turn-helix family protein [*Bacillus cereus*]
WP_001057116.1	100	2.20E-29	cold-shock DNA-binding domain-containing protein [*Bacillus* anthracis|*Bacillus thuringiensis*]
WP_001190080.1	100	2.20E-83	Mutator mutT protein (7, 8-dihydro-8-oxoguanine- triphosphatase) [*Bacillus* anthracis|*Bacillus cereus*|*Bacillus* mycoides|*Bacillus thuringiensis*]
WP_000575919.1	100	5.30E-58	MULTISPECIES: stage V sporulation protein AE [Bacilli|*Bacillus* anthracis|*Bacillus cereus*|*Bacillus* mycoides|*Bacillus* sp.|*Bacillus thuringiensis*|*Bacillus weihenstephanensis*|*Bacillus wiedmannii*|*Streptococcus pneumoniae*|*Bacillus thuringiensis*]
WP_001039637.1	99.4	3.40E-201	MULTISPECIES: spore photoproduct lyase [*Bacillus*|*Bacillus* sp.|*Bacillus thuringiensis*]
WP_001169829.1	97.7	8.60E-280	deoxyribodipyrimidine photo-lyase [*Bacillus cereus*]
WP_033658047.1	100	5.00E-83	transcription factor YdeB [*Bacillus cereus*]
WP_000176368.1	100	1.20E-30	cold-shock domain family protein-related protein [*Bacillus cereus* G9241]
WP_062822451.1	100	1.20E-29	Cold shock protein-related protein [*Bacillus cereus*]

WP_001141569.1	100	5.20E-32	Protein GerPF, required for proper assembly of spore coat, mutations lead to super-dormant spore [*Bacillus cereus*|*Bacillus* mycoides|*Bacillus* sp.|*Bacillus thuringiensis*]
WP_001133508.1	100	1.80E-27	MULTISPECIES: small acid-soluble spore protein Tlp [*Bacillus cereus* group]
WP_001179147.1	100	2.00E-30	cold-shock DNA-binding domain protein [*Bacillus weihenstephanensis* KBAB4]
WP_000429483.1	100	3.60E-53	MULTISPECIES: PadR family transcriptional regulator [*Bacillus*|*Bacillus cereus*|*Bacillus* sp.|*Bacillus thuringiensis*|*Streptococcus pneumoniae*]
WP_002137359.1	100	2.70E-63	MULTISPECIES: MerR family transcriptional regulator [*Bacillus*|*Bacillus cereus*|*Bacillus* mycoides|*Bacillus* sp.]
WP_000812335.1	100	9.80E-66	MarR family transcriptional regulator [*Bacillus cereus*]
WP_000809334.1	100	1.00E-41	MULTISPECIES: membrane protein insertion efficiency factor YidD [*Bacillus*|*Bacillus cereus*|*Bacillus* sp.|*Bacillus thuringiensis*|*Bacillus wiedmannii*]
EEL42557.1	100	1.60E-104	ISBma2, transposase [*Bacillus cereus* Rock3-42]
EOO75196.1	91.9	2.50E-22	transposase, partial [*Bacillus cereus* BAG1O-1]
WP_002107121.1	98.7	1.60E-122	DNA-binding response regulator [*Bacillus cereus*]
SCN42022.1	99.5	0.00E+00	Spore coat protein A [*Bacillus cereus*]
WP_070129429.1	100	4.60E-113	TetR family transcriptional regulator [*Bacillus weihenstephanensis*]
WP_048526354.1	100	1.00E-77	CtsR family transcriptional regulator [*Bacillus cereus*|*Streptococcus pneumoniae*]
WP_001085206.1	99.8	8.30E-256	MULTISPECIES: DNA repair protein RadA [*Bacillus cereus*]
WP_000415794.1	100	1.30E-95	MULTISPECIES: transcription termination/antitermination protein NusG [Bacilli|*Bacillus* anthracis|*Bacillus* bombysepticus|*Bacillus cereus*|*Bacillus* sp.|*Bacillus thuringiensis*|*Bacillus weihenstephanensis*|*Bacillus wiedmannii*]
WP_001029884.1	100	3.10E-32	Bacterial Protein Translation Initiation Factor 1 (IF-1) [*Bacillus* anthracis|*Bacillus cereus*|*Bacillus* mycoides|*Bacillus* sp.|*Bacillus thuringiensis*|*Bacillus* toyonensis]
WP_000217509.1	100	8.60E-155	MULTISPECIES: stage 0 sporulation protein [*Bacillus* anthracis|*Bacillus cereus*]
WP_000843036.1	100	2.30E-43	transition state transcriptional regulatory protein AbrB [*Bacillus cereus*|*Bacillus thuringiensis*|*Bacillus thuringiensis*]
WP_000070632.1	100	1.20E-20	small acid-soluble spore protein F (minor alpha/beta-type SASP) [*Bacillus* anthracis|*Bacillus* sp.]
WP_000648310.1	100	4.30E-91	Stage V sporulation protein T, AbrB family transcriptional regulator (SpoVT) [*Bacillus cereus*|*Bacillus thuringiensis*|*Bacillus* toyonensis]
WP_000372704.1	100	8.80E-112	Redox-sensitive transcriptional regulator (AT-rich DNA-binding protein) [*Bacillus cereus*|*Bacillus* mycoides|*Bacillus weihenstephanensis*]

Table S11. Genes analysis for various stress resistance of *B. cereus* SCL10 in classification.

Classification	Subject ID	Genes	Description	Number	Source
**cellular processe**	A9VMG8	*gpsB*	Cell cycle protein GpsB	1	Swiss-Prot
	2.A.6	RND Superfamily	The Resistance-Nodulation-Cell Division (RND) Superfamily	2	TCDB
	CL0551.1	*bcl*-2	Bcl-2 inhibitors of programmed cell death	18	Pfam
**Transporter**	1.A.22	MscL Family	The Large Conductance Mechanosensitive Ion Channel (MscL) Family	2	TCDB
	1.A.34	GJ-CC Family	The *Bacillus* Gap Junction-like Channel-forming Complex (GJ-CC) Family	2	
	2.A.41	CNT Family	The Concentrative Nucleoside Transporter (CNT) Family	7	
	2.A.88	VUT/ECF Family	Vitamin Uptake Transporter (VUT or ECF) Family	2	
	2.A.9	Oxa1 Family	The Cytochrome Oxidase Biogenesis (Oxa1) Family	2	
**Cold/salt resistance**	P62169	*cspC*	Cold shock-like protein CspC	1	Swiss-Prot
	Q45097	*cspB*	Cold shock-like protein CspB	1	
	Q81QK2	*cspE*	Cold shock-like protein CspE	1	
	9.B.12	SHP Family	The (Salt or Low Temperature) Stress-induced Hydrophobic Peptide (SHP) Family	1	TCDB
**oxidative stress resistance**	O34762	*ohrA*	Organic hydroperoxide resistance protein OhrA	1	Swiss-Prot
	O34777	*ohrR*	Organic hydroperoxide resistance transcriptional regulator	1	
	Q81TR6	*spx* 1	Regulatory protein Spx 1	1	
	Q9K813	*tpx*	Thiol peroxidase	1	
	WP_026594348.1	/	thiol reductase thioredoxin	1	GIS011
**Transcriptional regulation**	A9VQG4	*rex*	Redox-sensing transcriptional repressor Rex	1	Swiss-Prot
	A9VT66	*codY*	GTP-sensing transcriptional pleiotropic repressor CodY	1	
	B7IXH3	*nusB*	Transcription antitermination protein NusB	1	

	C3P4Y3	*hfq*	RNA-binding protein Hfq	1	
	C3PAG9	*nrdR*	Transcriptional repressor NrdR	1	
	WP_000421293.1	*codY*	GTP-sensing transcriptional pleiotropic repressor CodY	1	GIS004
DNA protection	A0R883	*recF*	DNA replication and repair protein RecF	1	Swiss-Prot
	A0RH75	*lexA*	LexA repressor	1	
	A9VHS5	*recO*	DNA repair protein RecO	1	
	C1ER74	*noc*	Nucleoid occlusion protein	1	
	C3LFM1	*uvsE*	UV DNA damage endonuclease	1	
	C3P9M9	*disA*	DNA integrity scanning protein DisA	1	
	P37572	*radA*	DNA repair protein RadA	1	
	Q8RPQ1	*dps*1	DNA protection during starvation protein 1	1	
	Q8RPQ2	*dps*2	DNA protection during starvation protein 2	1	
	3.A.11	DNA-T Family	The Bacterial Competence-related DNA Transformation Transporter (DNA-T) Family	4	TCDB
	3.A.12	S-DNA-T Family	The Septal DNA Translocator (S-DNA-T) Family	2	
	CL0382.3	/	DNA breaking-rejoining enzyme superfamily	10	Pfam
	WP_001039637.1	/	MULTISPECIES: spore photoproduct lyase	1	GIS009
	WP_001169829.1	/	deoxyribodipyrimidine photo-lyase	1	GIS009
Special Functions	3.A.9	CEPT or Tic-Toc Family	The Chloroplast Envelope Protein Translocase (CEPT or Tic-Toc) Family	2	TCDB

Figure Legends

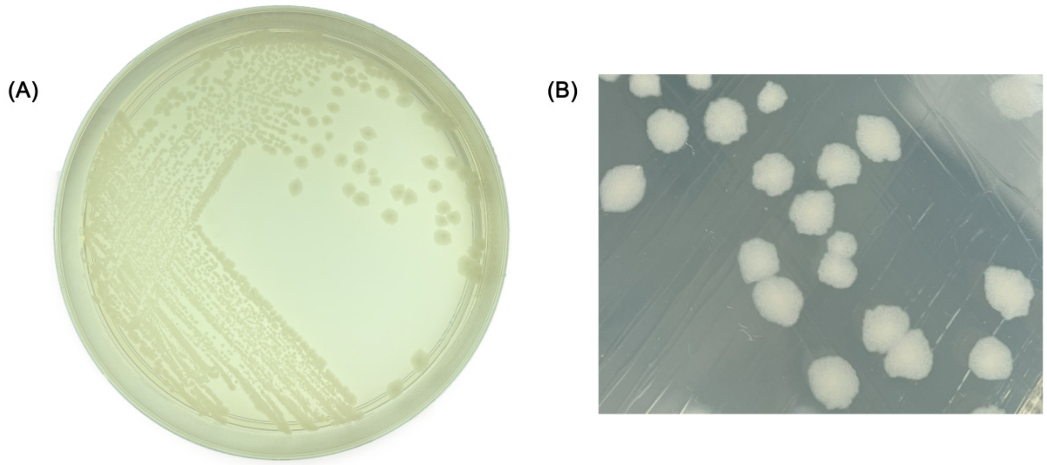

Figure S1. The colony morphology of *B. cereus* SCL10. (A) Quadrate section streak plate method for *B. cereus* SCL10 isolation on nutrient broth plate. (B) A closer view of the single colony of *B. cereus* SCL10.

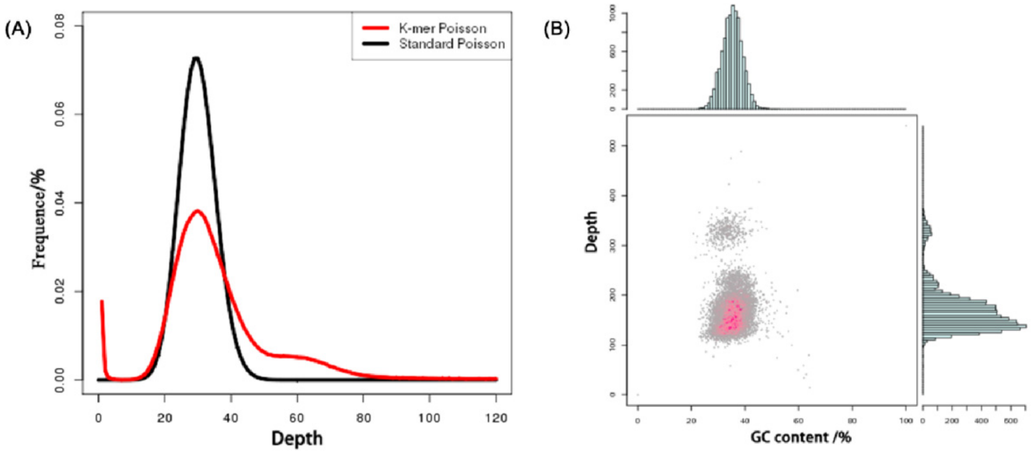

Figure S2. Data validity analysis of the whole genome of *B. cereus* SCL10. (A) K-mer=15 Depth-Frequence distribution. The X-axis indicates the K-mer depth, and the Y-axis indicates the ratio of the frequency at each depth to the total frequency. The red curve is the 15-mer depth distribution curve of the sequencing data, and the black curve is the standard Poisson curve that is closest to it. (B) GC content and depth of reads coverage distribution. The upper side is the GC content distribution and the right side is the sequencing depth distribution.

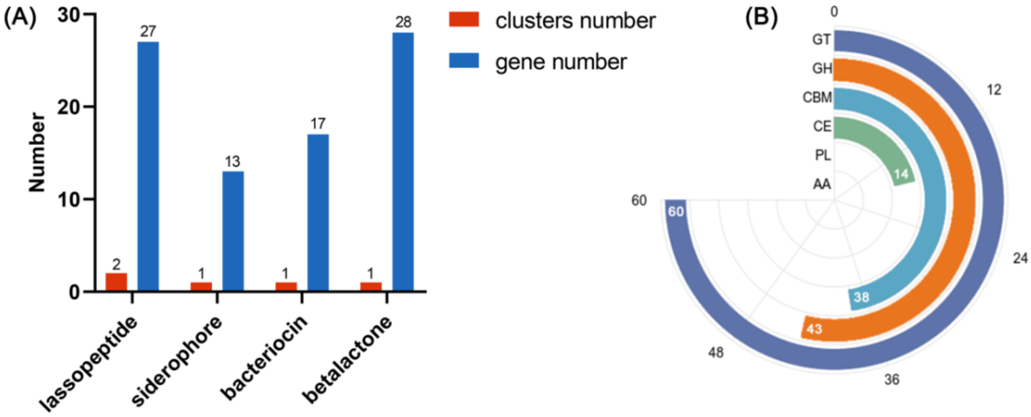

Figure S3. Gene clusters and CAZy annotation of *B. cereus* SCL10. (A) Distribution of gene clusters. (B) The classification and gene numbers of CAZy. The colors and values from the outer circle to the inner circle represent the different enzymes. CAZy: carbohydrate-active enzymes, GT: glycosyl transferase, GH: glycosidase hydrolase, CBM: carbohydrate-binding module, CE: carbohydrate esterase. PL: polysaccharide lyase, AA: auxiliary activity.

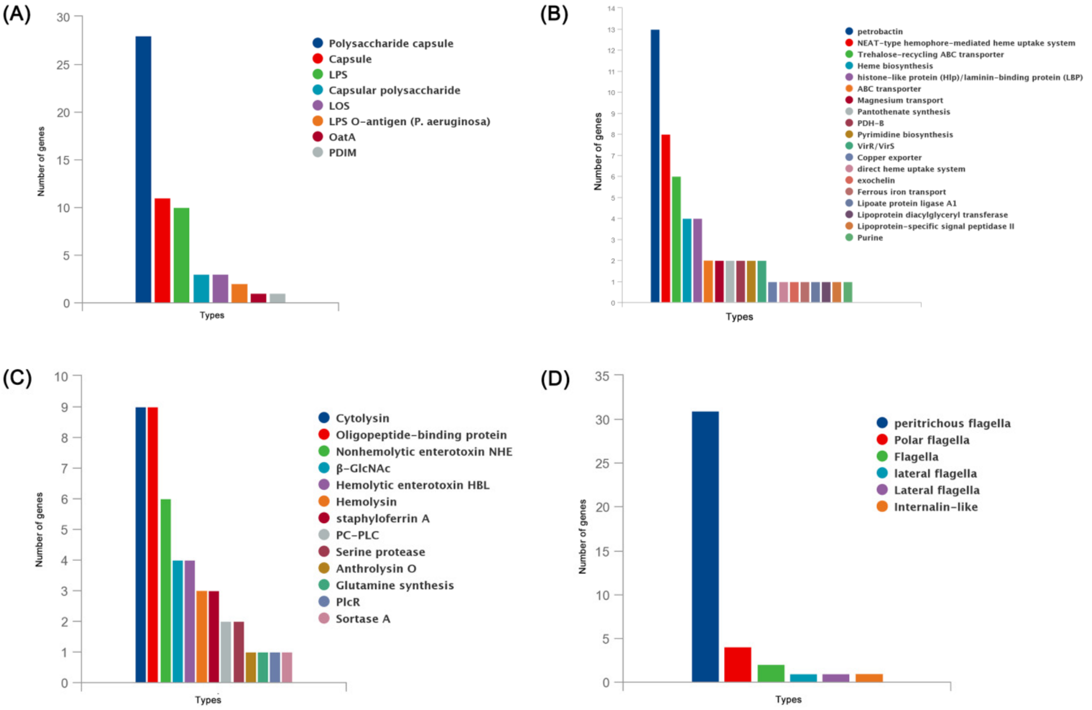

Figure S4. Distribution of *B. cereus* SCL10 virulence factors. (A) Distribution of immune modulation virulence factor. (B) Distribution of nutritional/metabolic virulence factor. (C) Distribution of exotoxin virulence factor. (D) Distribution of motility virulence factor.
